# Xanthogranulomatous pyelonephritis in a child with autoimmune joint disease

**DOI:** 10.1007/s00467-024-06582-4

**Published:** 2024-11-06

**Authors:** Pieter Willem Kriek, Maria Karsas, Jeané Cloete

**Affiliations:** Steve Biko Academic Hospital, Department of Paediatrics, School of Medicine, University of Pretoria, Pretoria, South Africa

**Keywords:** Xanthogranulomatous pyelonephritis, Kidney masses, *Escherichia coli*, Juvenile idiopathic arthritis, Child

## Abstract

Xanthogranulomatous pyelonephritis is a rare condition in paediatric patients, mostly described in middle-aged female patients. We present a 7-year-old female with juvenile idiopathic arthritis, who was found to have a kidney mass with a concurrent *Escherichia coli* urinary tract infection. Surgical excision was done out of concern for possible malignancy. Histology confirmed the diagnosis of xanthogranulomatous pyelonephritis and persistent *E. coli* infection.

## Case presentation

A 7-year-old African female from a rural South African community presented with a 1-year history of joint pain and swelling, associated with fever, which worsened 2 weeks prior to presentation. Previous treatment was limited to analgesia. Past medical history was uneventful, and she had no significant disease exposures. Her family had limited resources and poor socioeconomic status.

On examination she was normotensive, febrile (37.8 °C), and tachycardic (169 bpm). She was underweight-for-age (WHO Z-score: − 4.29), stunted (WHO *Z* score: − 3.86), her BMI-for-age plotted normal (BMI: 13.2; WHO *Z* score: − 1.94) despite appearing wasted. She had pallor, peripheral and periorbital oedema, and generalised lymphadenopathy.

Systemic examination revealed joint swelling, with limited range of movement, involving the metacarpophalangeal joints of the hands, both wrists, elbows, and knees. Her apex beat was displaced (sixth intercostal space, midaxillary line), and she had a non-radiating 2/6 ejection systolic murmur in the left parasternal area. Bilateral crackles were auscultated over the lungs. Her abdomen was distended, with tenderness to palpation and a palpable hepatomegaly (span 16 cm), with no ascites. Due to patient discomfort, it was difficult to palpate the kidneys.

Laboratory results (refer to Table 1 for detailed values) showed significant leucocytosis, normocytic hypochromic anaemia, a normal reticulocyte response, and thrombocytosis. She had markedly raised inflammatory markers, a wide globulin fraction, and an elevated lactate dehydrogenase with a normal uric acid. Antibody testing showed raised total immunoglobulins; auto-antibody testing was normal other than a weakly positive anti-nuclear antibody screen and positive anti-double stranded DNA antibody, with a raised lupus anticoagulant (61.20 s, ratio 1.81). Her complement classical pathway was normal, but she had a raised C3 and C4, with a normal anti-Cq1.

**Table 1:** Laboratory values

Laboratory test	Patient value on admission	Patient value on follow-up*	(Population specific reference range) units used
Full blood count:			
Haemoglobin	6.1	13.4	(10.7–15.1) g/dL
Haematocrit	21.8	43.3	(32–45) %
Mean corpuscular volume	88.3	82.2	(77.1–91.5) fL
Red cell distribution width	19.2	17.2	(11.6–14.8) %
Platelets	655	577	(180–440) × 10^9^/L
White cell count	18.43	11.85	(3.90–10.20) × 10^9^/L
Neutrophils	9.47	5.21	(1.70–5.00) × 10^9^/L
Lymphocytes	7.34	5.66	(1.90–4.30) × 10^9^/L
Monocytes	1.36	0.64	(0.00–0.80) × 10^9^/L
Eosinophils	0.02	0.17	(0.00–0.70) × 10^9^/L
Serum kidney function and electrolytes:			
Sodium	136	135	(136–145) mmol/L
Potassium	5.2	4.5	(3.4–4.7) mmol/L
Chloride	104	103	(98–107) mmol/L
Bicarbonate	14	16	(23–29) mmol/L
Urea	4.0	3.0	(1.4–5.7) mmol/L
Creatinine	41	45	(30–48) mmol/L
Calcium	2.14	2.55	(2.12–2.57) mmol/L
Magnesium	0.86	0.87	(0.66–0.95) mmol/L
Phosphate	1.57	1.62	(1.00–1.80) mmol/L
Inflammatory markers:			
ESR**	> 100	80	(< 16) mm/h
C-reactive protein	154	2	(< 10) mg/L
Liver enzymes and function:			
Total protein	97	86	(57–80) g/L
Albumin	24	41	(29–42) g/L
Total serum bilirubin	10	3	(5–21) umol/L
Conjugated bilirubin	7	< 2	(0–5) umol/L
Aspartate transaminase	50	24	(0–41) U/L
Alanine transaminase	24	29	(5–25) U/L
Alkaline phosphatase	224	228	(69–325) U/L
Gamma-glutamyl transferase	285	34	(4–22) U/L
Lactate dehydrogenase	646	592	(110–295) U/L
Uric acid	0.17	0.23	(0.12–0.30) mmol/L
Iron studies			
Iron	6.0	7.3	(9.0–21.5) umol/L
Transferrin	2.07	2.77	(1.77–3.71) g/L
Transferrin saturation	12	11	(15–50) %
Ferritin	1086	224	(6–216) ug/L
Vitamin B12	1237		(218–1190) pmol/L
Folate	17.7		(> 29.7) nmol/L
Antibodies:			
Total IgG	31.80	17.20	(4.85–14.73) g/L
Total IgA	2.44	2.21	(0.28–1.80) g/L
Total IgM	2.61	3.13	(0.30–1.65) g/L
Rheumatoid factor	12		(< 16) IU/mL
Anti-nuclear antibody	Positive 7.5	Negative 0.5	ratio
Anti-ds-DNA antibody**	Positive 198.0	Negative 7.5	IU/mL
Complement:			
Classical pathway	920	233	(392–1019) CH100 units
C3	2.70	2.75	(0.90–1.80) g/L
C4	0.73	1.31	(0.10–0.40) g/L
Anti-Cq1	4	2	(< 10) RU/mL
Urine analysis:			
Protein	0.29	0.32	g/L
Creatinine	8.2	9.8	mmol/L
Protein:creatinine ratio	0.35	0.033	(< 0.15 = A1 normal to mildly increased 0.015–0.050 = A2 moderately increased > 0.050 = A3 severely increased > 0.200 = nephrotic range)*** g/mmolcreat

^*^Follow-up values are from 13 weeks after admission

^**^ESR Erythrocyte sedimentation rate, *Anti-ds-DNA antibody* anti-double-stranded-DNA antibody

^***^Kidney Disease: Improving Global Outcomes (KDIGO) reference ranges for proteinuria

Infectious disease screening was negative for HIV, tuberculosis, viral hepatitis, and cytomegalovirus. Her Epstein Barr virus viral load was elevated (27,200 copies/mL, log conversion 4.4). Blood cultures were negative. Urine analysis demonstrated nephrotic range proteinuria, sterile pyuria on three separate specimens, and another cultured *Escherichia coli* with extended beta-lactamase that was sensitive to carbapenems and aminoglycosides. Out of concern for possible malignancy flow cytometry, a lymph node biopsy, urine homovanillic acid, bone marrow aspiration, and trephine biopsy were performed, which only showed reactive features.

**Fig. 1:**
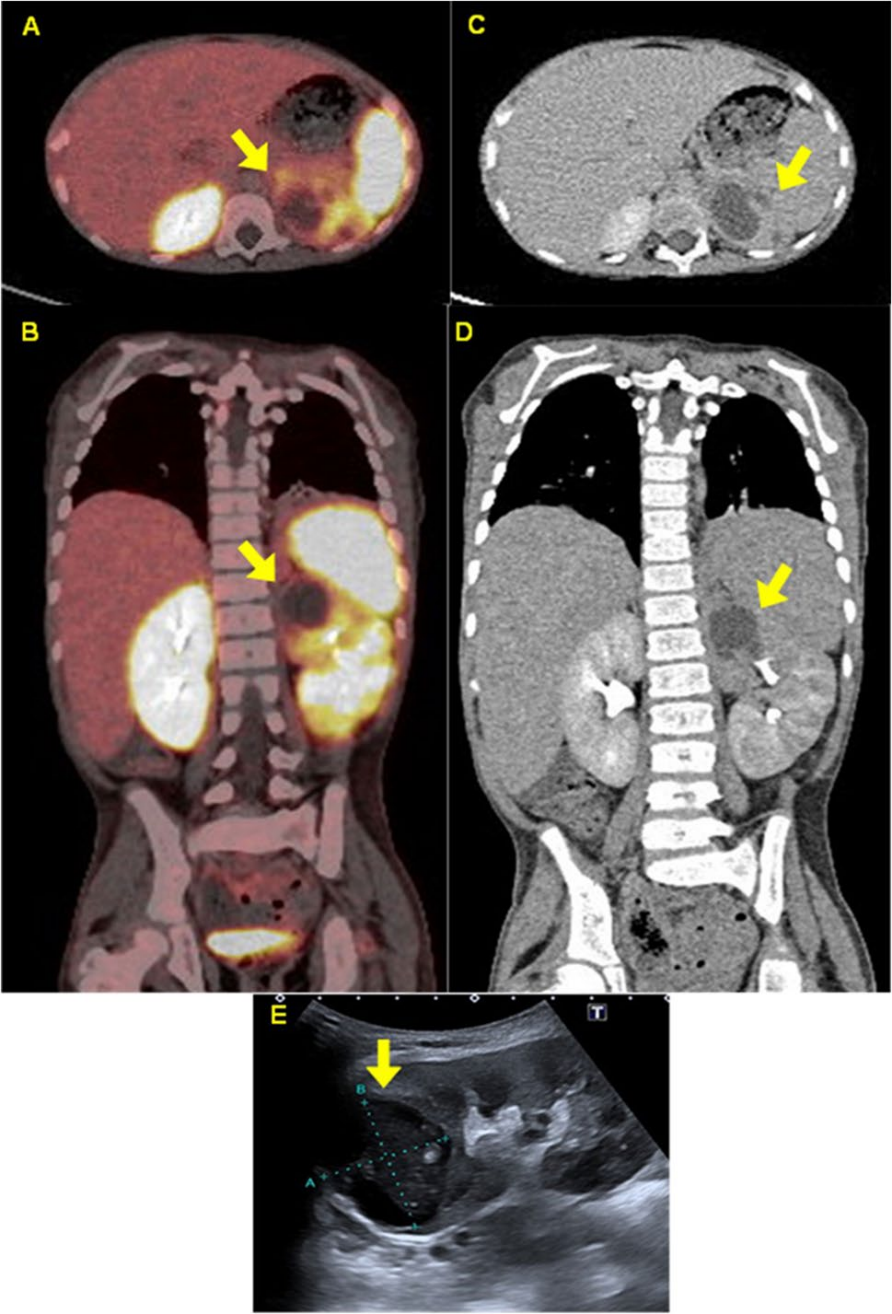
This series of images demonstrates the XGP (arrows) on axial (**A**) and coronal (**B**) views of the Ga-68-DOTATE positron emission tomography, axial (**C**) and coronal (**D**) views of the computerised tomography, and an ultrasound image (**E**). Note the lower uptake of tracer in the affected kidney, pronounced hepatomegaly, and the fusion of some of the vertebrae

Imaging studies included an abdominal ultrasound that identified a complex cystic mass in the upper pole of the left kidney and bilaterally enlarged kidneys (right kidney: 9.3 × 4.3 cm; left kidney: 9.4 × 4.4 cm). Computed tomography confirmed these findings, revealing a cystic mass measuring 3.8 × 3.4 × 3.15 cm with delayed enhancement and containing homogeneous fluid (10–12 Hounsfield units). Ga-68-DOTATATE positron emission tomography showed low-grade tracer uptake by the kidney mass, accumulation in joints, and normal excretion by the kidneys (see Fig. 1).

Our working diagnosis included an autoimmune joint disease, possibly systemic lupus erythematosus (SLE) or juvenile idiopathic arthritis (JIA), accompanied by a kidney mass. Malignancy was considered less likely. Management included adequate analgesia and antibiotic therapy for the urinary tract infection (UTI). Rheumatological treatment involved pulses of methylprednisolone and mycophenolic acid.

Surgical intervention was undertaken; intraoperatively pus was drained from the cystic lesions that cultured *Escherichia coli* with sensitivity to penicillin. An attempt at left partial nephrectomy of the upper pole was made but was converted to a total nephrectomy due to uncontrolled bleeding.

Histopathological examination showed gross and microscopic features of xanthogranulomatous pyelonephritis (XGP): a tan-brown–coloured specimen showed an ulcerated renal pelvis lined by granulation tissue, obliteration of the renal sinus, and tubules by inflammatory tissue. The renal parenchyma was replaced by foamy histiocytes, lymphocytes, and plasma cells. Foamy macrophages could be seen in the collecting system and medulla, with sparing of the renal cortex. There were features of interstitial fibrosis with foci of interstitial lymphocytes and plasma cells.

The patient recovered well after surgery and on later follow-up was well controlled on steroid sparing agents. Repeat auto-antibody screening was all negative which made systemic JIA our primary diagnosis for this patient. She has not had further UTIs and continues to enjoy a quality of life comparable to her premorbid state (see Table 1 for repeat laboratory values).

## Discussion

XGP typically follows a chronic kidney infection or obstruction resulting in a destructive granulomatous inflammation. Patients usually present with symptoms of UTI, flank pain, and sometimes with a flank mass [1]. It is more frequent in middle aged women (70% being female) but can occur at any age. *E. coli* and *Proteus mirabilis* are most frequently cultured [1]. Gross pathology typically shows an enlarged kidney with a staghorn-like appearance and a tan-brown or yellow colour. It is not uncommon to find renal calculi. Histology findings of renal parenchyma replaced by foam cells, and xanthogranulomatous nodules rimmed by foamy macrophages and a fibroblastic response are typical. The only curative treatment is a nephrectomy paired with empiric antibiotic therapy [1, 2].

Since the first paediatric cases were described in 1963, fewer than 350 paediatric cases have been published in the literature and mostly consist of case series and case reports. The largest paediatric case series was described in a single-centre study by Stoica et al. [2]. This series highlights important differences found in the paediatric cohort: a male predominance; focal disease was less common; statistically significant findings of anaemia, thrombocytosis, raised inflammatory markers, and hypomagnesemia [2]. Our patient shared these findings, except for sex and her serum magnesium was normal. Pre-existing comorbid disease such as chronic inflammation, immunocompromise, partially treated urosepsis, and urinary obstruction were mentioned as important risk factors [2]. In our patient, these factors are associated with JIA and could have contributed to XGP.

In the literature, there are only two other cases of XGP that also had pre-existing rheumatological disease in children; one was a 17-year-old female with treated SLE, and the other was a 14-year-old male who had been treated for multisystem inflammatory syndrome in children [3, 4]. Amyloidosis has been described as an important and increasing morbidity in the diagnosis of XGP; this does tend to still be present in the remaining kidney after a nephrectomy has been done of the one with the mass [2]. This offers a possible explanation for our patient’s enlarged kidneys. It might also be explained by the diagnosis of systemic JIA.

Another case report of a 7-year-old male patient that developed anti-glomerular basement membrane disease 3 months after nephrectomy for XGP, posed the question whether autoimmune disease could be triggered by XGP—especially in certain HLA-subtypes [5]. This would be relevant in our patient as her JIA and XGP were diagnosed concurrently.

## Summary

According to our knowledge, this is the first paediatric case of XGP in sub-Saharan Africa to be published. This is also the first published case of XGP with a comorbid diagnosis of JIA. XGP should be an important diagnosis to consider in patients with a UTI and a kidney mass, as it is still mostly diagnosed retrospectively on histology. The question remains whether autoimmune or rheumatological disease is a contributing risk factor to the pathogenesis of XGP or vice versa. Further research would be needed on the topic to examine a possible bidirectional pathogenesis.

### What is new?

- This is the first published case XGP with a comorbid diagnosis of JIA, and the first paediatric case published in sub-Saharan Africa.
